# Differences in self-perceived general health, pain, and depression 1 to 5 years post-stroke related to work status at 1 year

**DOI:** 10.1038/s41598-020-70228-2

**Published:** 2020-08-06

**Authors:** Emma Westerlind, Hanna C. Persson, Annie Palstam, Marie Eriksson, Bo Norrving, Katharina S. Sunnerhagen

**Affiliations:** 1 Department of Clinical Neuroscience, Institute of Neuroscience and Physiology, Sahlgrenska Academy, University of Gothenburg, and Sahlgrenska University Hospital, Gothenburg, Sweden; 2 Department of Statistics, USBE, Umeå University, Umeå, Sweden; 3 Department of Clinical Sciences, Section of Neurology, Lund University, Skåne University Hospital, Lund, Sweden

**Keywords:** Cerebrovascular disorders, Stroke, Occupational health

## Abstract

Stroke is one of the most common diseases and has several potential consequences, such as psychological problems and pain. Return to work (RTW) after stroke in working-age individuals is incomplete. The present study aimed to investigate differences in self-perceived general health, pain, and depression between 1 and 5 years post-stroke related to RTW status. The study was nationwide, registry-based and the study population (n = 398) consisted of working-age people who had a stroke in 2011 and participated in 1-year and 5-year follow-up questionnaire surveys. Shift analyses with the Wilcoxon signed rank test and logistic regression were used. RTW within the first year post-stroke was associated with better self-perceived general health, less pain, and less depression both at 1 and 5 years post-stroke, compared with the no-RTW group. However, the RTW group had significant deterioration in general health and pain between 1 and 5 years, while the no-RTW group had no significant change. RTW was a significant predictor of lower odds of improvement in general health and pain between 1 and 5 years. This emphasizes the need for continued follow-up and support to ensure a balance between work and health for RTW individuals after stroke.

## Introduction

Globally, stroke is the second largest cause of death and the second most common cause of disability^1^. Although the total incidence of stroke is decreasing in most parts of the world, the number of disability adjusted life years is increasing^1^, as is the incidence of stroke in the working age population (20–64 years old)^2^. Consequences of stroke, in addition to physical and cognitive impairment, include depression^3^ and pain^4^. The prevalence varies, but it has been estimated that approximately 1 of 3 suffers from depression^3^ and up to half of the people have pain post-stroke^4^. Ten years post-stroke, pain and psychological problems are two of the symptoms still experienced by a substantial proportion of people with stroke^5^.

Hypothetical recovery trajectories after stroke show a fast recovery during the first weeks and plateaus after approximately 6 months. After the initial 6 months the patterns are heterogeneous, with some people declining and some continuing to improve^6^. In line with this, improvements in activities of daily living the first year post-stroke has been reported, but between 1 and 3 years post-stroke there seems to be a small decline^7^. Participants in another study reported less self-efficacy, less optimism and less proactive coping 2 years post-stroke compared with 2 months post-stroke^8^. A decline in several self-reported functional aspects such as strength, activities of daily living, and mobility between 1 and 6 years post-stroke has also been reported^9^.

Absence from work due to sickness could have an effect on the individual’s life situation as a whole. In addition to its impact on the financial situation, being on long-term sick leave is detrimental to psychological well-being and sleep^10^, and is even a risk factor for developing stroke^11^. Working seems to be important for health^12^ and for building a personal identity^13^.

In stroke, the sequelae often influence the affected person’s ability to work. According to a review, the rate of return to work (RTW) after stroke varies between 7 and 75%, among different countries with a median RTW of 53% within 1 year post-stroke^14^. RTW has been associated with higher well-being and life satisfaction after stroke^12^. The people that had RTW after stroke reported less depression and better quality of life than the people who had not RTW^15^. The full impact of RTW on several aspects of health is however yet unknown. Furthermore, there is lack of research investigating the role of RTW in the overall long-term recovery after stroke.

### Aim

The aim was to investigate differences in self-perceived general health, pain, and depression between 1 and 5 years post-stroke in people who have RTW compared with people who have not RTW after a stroke.

## Methods

### Study design and population

The present study is an observational registry-based study. The included participants were from the Riksstroke (the Swedish Stroke Register) with a stroke in 2011. Further inclusion criteria were diagnosis code I61 (intracerebral haemorrhage), I63 (ischemic stroke) or I64 (unspecified stroke) according to the international classification of diseases (ICD-10) codes; being 18–58 years of age at the time of stroke; and participation in both 1-year and 5-year follow-up questionnaire surveys by Riksstroke. Exclusion criteria were being registered with a previous stroke; living at a nursing home at the time of stroke onset; being registered with sickness compensation of more than 50% 1 year prior to the stroke; or dying before the 5 year follow-up.

### Data collection

Registries from Riksstroke, the Social Insurance Agency, Statistics Sweden, and the National Board of Health and Welfare were linked in the present study by using Swedish personal identification numbers. The initial linkage was performed at the National Board of Health and Welfare and anonymised data were delivered to the researchers.

The study population originated from Riksstroke, which also contributed stroke-related and demographical data, as well as the follow-up questionnaires. Riksstroke is a national quality registry that has a coverage rate of > 90% of all people having a stroke and being treated at a hospital in Sweden^16^, and includes all hospitals admitting acute stroke patients. Riksstroke has a 1-year follow-up questionnaire survey sent out by post to all surviving people in the registry, examining a variety of items concerning life after stroke. An additional 5-year follow-up questionnaire was sent to all surviving people registered with a stroke during 2011. Non-responders received two remainders by post.

The Swedish Social Insurance Agency is a public authority through which people of all occupations, parental leave or unemployment, are eligible for benefits when on sick leave. It provided sickness absence data up to 5 years post-stroke to the study. Sickness absence can be covered with either sickness benefit (sick-leave) or sickness compensation (early retirement) for 25%, 50%, 75% or 100% of full-time. For the first 2 weeks of absence from work, the employer pays sickness pay, and thereafter the Social Insurance Agency pays sickness benefit. Sickness compensation is an alternative when RTW is unlikely, due to sickness.

Socioeconomic data was gathered from Statistics Sweden, which covers people registered in Sweden.

The National Board of Health and Welfare provided data about if and when a participant had died during the study period.

### Variables

Level of consciousness at admission to hospital according to the Reaction Level Scale (RLS)^17^ was used as an indicator for stroke severity. Level of consciousness has been used successfully as a proxy for stroke severity^18^. The levels used were: alert (RLS 1), drowsy (RLS 2–3) and unconscious (RLS 4–8).

Educational level from Statistics Sweden was classified in four levels: primary school (≤ 9 years), secondary school (10–12 years), short university education (13 years) and long university education (≥ 14 years).

Country of birth was obtained from Statistics Sweden and presented as Sweden, Nordic countries (except for Sweden), European countries (except for the Nordic countries), or countries outside of Europe.

Work status was defined according to sickness absence data from the Social Insurance Agency. RTW was defined as not being registered with more than 50% sickness benefit or sickness compensation for at least 2 months. For the analyses, RTW within 1 year (365 days) was used as the variable RTW.

To assess self-perceived general health, pain, and depression, the 1-year and 5-year follow-up questionnaire surveys by Riksstroke were used. The participants had to answer the questions in both the 1-year and 5-year follow-ups for each subject to be valid. The question *How would you assess your general health?* was answered with *Very good*, *Quite good*, *Quite poor*, or *Very poor.* The question *Do you have any pain?* was answered with *Never or almost never*, *Sometimes*, *Often*, or *Constantly.* The question *Do you feel depressed?* was answered with *Never or almost never*, *Sometimes*, *Often*, or *Constantly*.

### Statistical methods

IBM SPSS 25 was used to store and analyse data. The significance level was set at an alpha of 5% and the tests were two-tailed. For comparisons between groups, the Fischer’s exact test and the Mann–Whitney U test were used.

Shift analyses were performed to assess change in self-perceived general health, pain, and depression between 1 and 5 years post-stroke. The analyses were divided according to RTW groups at 1 years post-stroke. The shifts were graphically presented with bar graphs. The Wilcoxon signed rank test was used to statistically analyse the shifts.

Logistic regression was used to analyse if RTW 1 year post-stroke could predict improvement in self-rated general health, pain, and depression between 1 and 5 years post-stroke. Three different models were used for each of the dependent variables, which were dichotomized into *improved rating*, and *unchanged or deteriorated rating*. RTW was the independent variable and adjusted for stroke severity, age, and sex in all models. The Hosmer–Lemeshow test was used to assure goodness of fit for the models.

### Ethical considerations

The Regional Ethical Review Board in Gothenburg approved the study in 2017 (Dnr922-17). No written or verbal informed consent was obtained from the participants, but the participants were informed that the data in Riksstroke could be used for research and that they could withdraw their participation in the registry at any time. Furthermore, to participate in the follow-up questionnaires was voluntary. According to the Swedish Data Inspection Board, quality registries are exempt from requirement for informed consent because they provide improvements in the quality of care and treatment that are of general interest.

## Results

### Inclusion and characteristics of the participants

**Figure 1:**
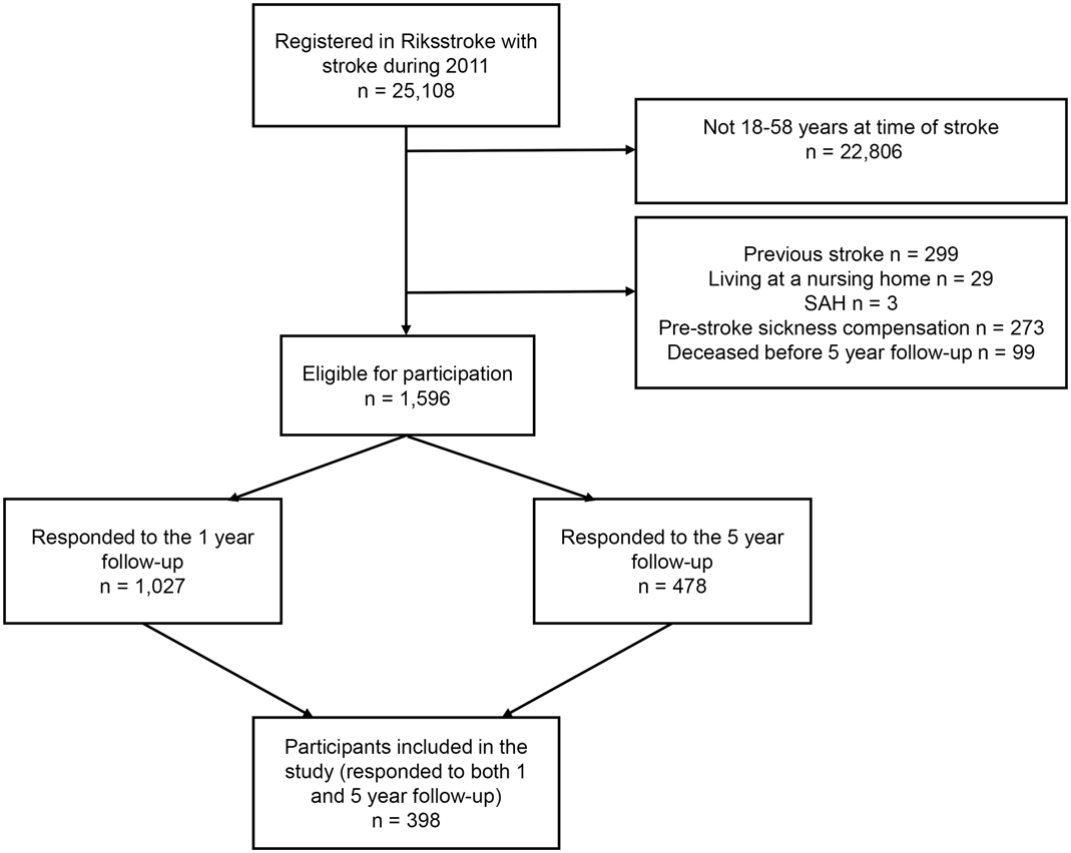
The inclusion of participants. Abbreviations: SAH: subarachnoid haemorrhage.

A total of 398 (24.9%) participants of the 1596 eligible people fulfilled the inclusion criteria by responding to both the 1-year and 5-year follow-up questionnaires and were therefore included in the study (Fig. 1). There was no significant difference between the non-participants who did not respond to both of the surveys and the participants who did respond regarding sex (p = 0.951), stroke type (p = 0.889), stroke severity (p = 0.355), educational level (p = 0.153), or RTW status (p = 0.497). However the study participants were significantly older than the non-responders (p < 0.001).

A total of 1,027 (64.3%) responded to the 1-year follow-up. They had a significantly less severe stroke (0.021) and were older (p = 0.001) than the non-responders. There was no significant difference in sex (p = 1.000), stroke type (p = 0.273), educational level (p = 0.085), or RTW status (p = 0.426). There were 478 (29.9%) people who responded to the 5-year follow-up. They were significantly older (p < 0.001) than the non-responders but there was no significant difference in sex (p = 0.645), stroke type (p = 0.952), stroke severity (p = 0.383), educational level (p = 0.434), or RTW status (p = 0.124).

As seen in Table 1, the mean age of the participants was 50 years and the majority (66%) were men. and 92% had the mildest stroke severity (alert in level of consciousness). Of the 398 participants, 298 (74.9%) had RTW within 1 year and 355 (89.2%) had RTW within 5 years.

**Table 1:** Characteristics of the participants, total and divided according to RTW status at 1 year post-stroke.

Characteristics	Total	RTW	No-RTW
Total, n	398	298	100
Age, mean (SD)	50.4 (7.45)	50.6 (7.20)	49.3 (8.08)
Sex, n (%)			
Men	263 (66.1)	203 (68.1)	60 (60.0)
Women	135 (33.9)	95 (31.9)	40 (40.0)
Educational level, n (%)			
Primary school	74 (18.6)	54 (18.1)	20 (20.0)
Secondary school	204 (51.3)	155 (52.0)	49 (49.0)
Short University education	27 (6.8)	19 (6.4)	8 (8.0)
Long University education	93 (23.4)	70 (23.5)	23 (23.0)
Country of birth, n (%)^a^			
Sweden	343 (86.8)	258 (87.2)	85 (85.9)
Nordic countries outside of Sweden	14 (3.5)	12 (4.1)	2 (2.0)
European countries outside of the Nordic countries	17 (4.3)	10 (3.4)	7 (7.1)
Countries outside of Europe	21 (5.3)	16 (5.4)	5 (5.1)
Stroke type, n (%)			
IS	331 (83.2)	261 (87.6)	70 (70.0)
ICH	65 (16.3)	35 (11.7)	30 (30.0)
Unspecified stroke	2 (0.5)	2 (0.7)	0 (0.0)
Level of consciousness, n (%)^b^			
Alert	358 (92.0)	282 (96.2)	76 (79.2)
Drowsy	25 (6.4)	10 (3.4)	15 (15.6)
Unconscious	6 (1.5)	1 (0.3)	5 (5.2)
Reperfusion treatment, n (%)			
Thrombolysis	49 (12.4)	34 (11.4)	15 (15.0)
Thrombectomy	8 (2.0)	5 (1.7)	3 (3.0)

^a^: n = 395. ^b^: n = 389. Abbreviations: IS: ischemic stroke; ICH: intracerebral haemorrhage; SD: standard deviation.

### Self-perceived general health, pain, and depression

The largest part of the participants experienced depression never or almost never, both at 1 year and 5 years post-stroke, and there was no significant change from 1 to 5 years (Table 2). A majority reported having pain never or almost never 1 year post-stroke, but a significant increase in pain was shown at 5 years. A small yet significant deterioration in self-rated general health could also be seen between 1 and 5 years post-stroke.

**Table 2:** Self-perceived general health, pain, and depression at 1 year and 5 years post-stroke, compared with the Wilcoxon signed rank test.

	1 year post-stroke	5 years post-stroke	P value
How would you rate your general health? n (%)^a^			0.034
Very good	94 (24.9)	74 (19.6)	
Fairly good	240 (63.7)	253 (67.1)	
Pretty bad	38 (10.1)	45 (11.9)	
Very bad	5 (1.3)	5 (1.3)	
Are you in pain? n (%)^b^			< 0.001
Never or almost never	205 (53.0)	149 (38.5)	
Sometimes	111 (28.7)	135 (34.9)	
Often	43 (11.1)	59 (15.2)	
Constantly	28 (7.2)	44 (11.4)	
Do you feel depressed? n (%)^c^			0.232
Never or almost never	169 (43.2)	180 (46.0)	
Sometimes	166 (42.5)	166 (42.5)	
Often	50 (12.8)	35 (9.0)	
Constantly	6 (1.5)	10 (2.6)	

^a^: n = 377. ^b^: n = 387. ^c^: n = 391.

### Impact of RTW on general health, pain, and depression

**Figure 2:**
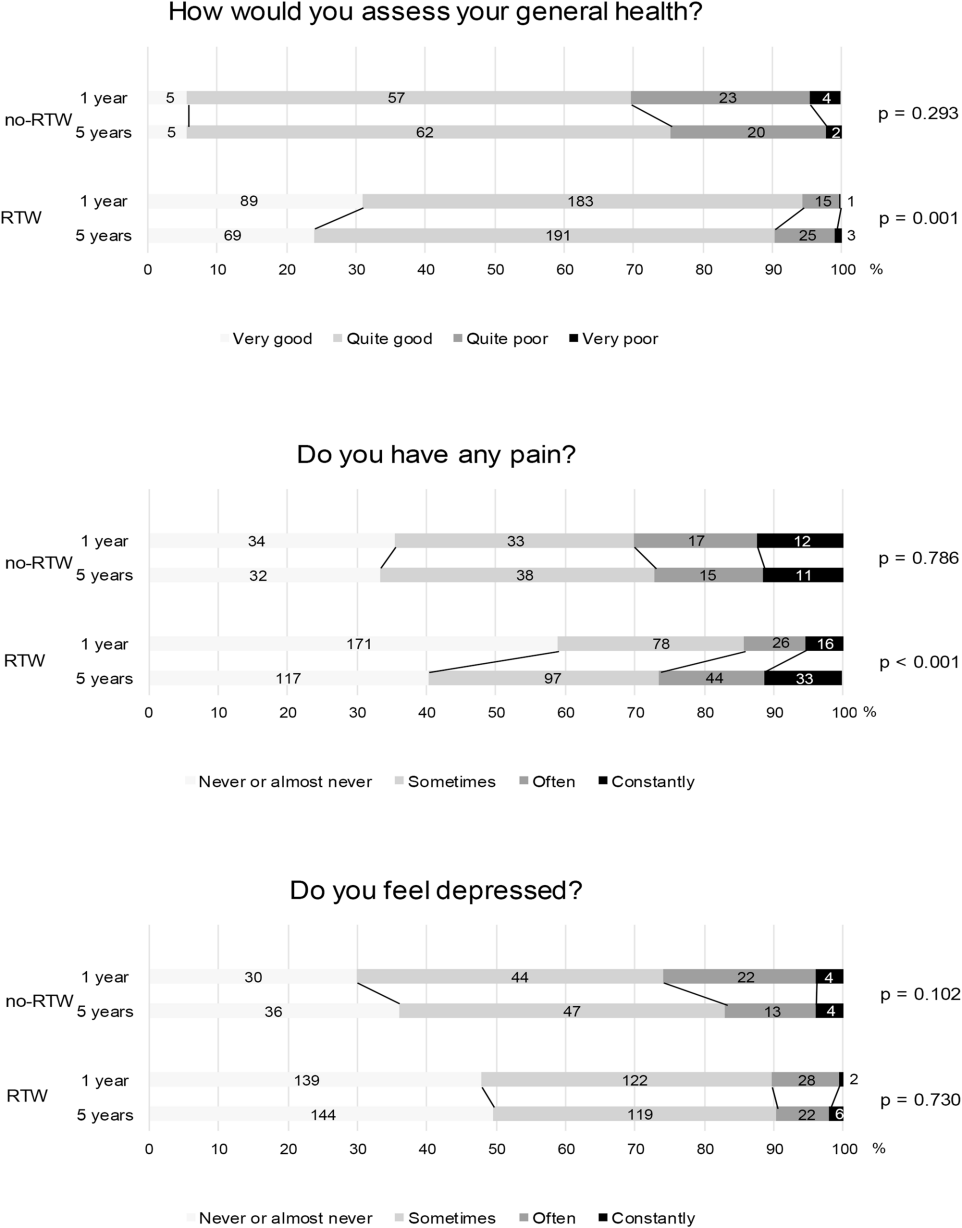
Shifts of proportions of self-reported general health, pain, and depression between 1 and 5 years post-stroke, comparing the no-RTW and RTW groups after 1 year. The results were analysed using the Wilcoxon signed rank test. Figures within bars represent number of respondents in each category. Abbreviations: RTW: return to work.

As seen in Fig. 2, the participants that had RTW within the first year experienced significantly better general health, and less depression both at 1 year and 5 years post-stroke compared with those not RTW. The RTW group also experienced significantly less pain at 1 year, and slightly but non-significantly less pain at 5 years. However, there was a significant shift towards lower general health and more pain between 1 and 5 years post-stroke in the RTW group. There was no significant change in depression. In the no-RTW group, there were no significant shifts in responses to any of the questions.

RTW within 1 year post-stroke (adjusted for stroke severity, age, and sex) significantly predicted improvement in general health and pain between 1 and 5 years after stroke (Table 3). No significant prediction was found in depression. The people that had RTW the first year had lower odds of an improvement in experienced general health from 1 to 5 years compared with the no-RTW group (OR 0.441). The RTW group also had lower odds of improvement in pain compared with the no-RTW group (OR 0.424).

**Table 3:** Logistic regressions modelling improvement in general health, pain, and depression between 1 and 5 years post-stroke. Odds ratios (OR) and 95% confidence intervals (95% CI) adjusted for stroke severity, age and sex.

Dependent variable	Independent variable	OR	95% CI	P value
Better general health^a^	RTW	0.441	0.224–0.869	0.018
Less pain^b^	RTW	0.424	0.221–0.813	0.010
Less depression^c^	RTW	0.685	0.389–1.207	0.190

^a^: n = 368. Hosmer–Lemeshow test p = 0.290 ^b^: n = 378. Hosmer–Lemeshow test p = 0.794. ^c^: n = 382. Hosmer–Lemeshow test p = 0.770. Abbreviations: RTW: return to work; OR: odds ratio; CI: confidence interval.

## Discussion

The people that had RTW within the first year after stroke had better self-perceived general health, less depression and less pain at both 1 and 5 years post-stroke, compared with the people that had not RTW. However, the RTW group had significant deterioration in general health and pain between 1 and 5 years, while the no-RTW group did not. Furthermore, RTW within 1 year was a significant predictor for lower odds of improvement in general health and pain between 1 and 5 years.

Better self-perceived general health, and less pain, and depression post-stroke in the RTW group compared with the no-RTW group are expected, and RTW has been associated with better well-being, quality of life, and less depression in previous research^12,15^. One explanation could be that the people who RTW after stroke usually have milder symptoms from stroke^19^. This is also seen in the present study where the RTW group had lower stroke severity than the no-RTW group.

The observed association between RTW at 1 year and a deterioration in self-perceived general health and pain between 1 and 5 years is a relatively new finding in quantitative studies. However, it is still clear that the RTW group experienced better health and fewer symptoms of depression and pain than the no-RTW group. The reason for the deterioration in the RTW group is unknown, and might indicate that the effects of work on health are not all positive. Interview studies have shown that people who have RTW after stroke and subarachnoid haemorrhage still struggle with symptoms, sometimes invisible, several years after stroke and RTW^20,21^. It has been shown that sickness presenteeism (working despite sickness) is associated with worse self-rated health, sickness absence in the future, and several symptoms such as neck pain and depression^22-24^. It has been suggested that this could result from emotional exhaustion^22^. There are work-related differences related to sickness presenteeism, with people working in the education sector and the care and welfare sector having the highest frequency^23^. There are also sex and age differences, with women and people of middle age tending to have more sickness presenteeism^23^. It could be speculated that the participants in the present study who managed to RTW might have received less follow-up by the healthcare, and therefore did not continue to improve in the long-term. Previous studies have shown that people with stroke experience a lack of follow-up by the healthcare system and some feel abandoned after discharge, which is detrimental to their well-being negatively^21,25^. Perhaps a more structured long-term follow-up after stroke, regardless of initial improvement and RTW, would be beneficial for these people.

Regardless of work status, the total population in the present study showed an overall significant worsening in self-perceived general health and pain, and a slight but non-significant worsening in depression from 1 to 5 years post-stroke. Several studies have shown that the recovery after a stroke is not only increasing over time, but it can also decrease after the first improvement^7-9,26^. The fact that the participants were 4 years older at the 5-years follow-up compared with 1-year follow-up could affect their recovery; for example, older age is a risk factor for neuropathic pain^27^.

One limitation of the study that must be discussed is the low response rate to the follow-up surveys. The drop-out analyses showed no significant difference in sex, stroke severity, stroke type, educational level, or work status between the participants and the non-participants who did not respond to both the 1- and 5-year questionnaires. The participants were older, however, and it is likely that it is the young age of the present population that led to the lower response rate, but there is no indication from the results in the present study that the age distribution of the responders substantially affects the RTW status of the study population. A previous study that also used the Riksstroke population and follow-up questionnaires at 3 and 5 years post-stroke showed the same age pattern with people < 65 years old having a lower response rate than people ≥ 65 years of age^26^. According to published research, the response rate for questionnaire surveys is lower in younger than in older people^28^. Even if the drop-out analyses did not indicate it, the low response rate introduces a risk of selection bias that lowers the generalizability of the results. For future questionnaire surveys in a younger population, perhaps an electronic targeted questionnaire with would be more suitable than a general postal questionnaire.

The definition of RTW in the present study should also be discussed. RTW is defined according to registry data on social insurance due to sickness, which could differ for example from the self-reported data used in other studies. Registries enables the use of exact data without drop-outs, but this data is limited by the uncertainty as to whether all the participants in the RTW group actually have RTW. There is a risk that some of the participants counted as RTW are not working but instead receive money from next of kin or the Social Services in Sweden. Furthermore, general health, pain, and depression were assessed in only a general subjective sense in the present study. No objective or specific assessment tools other than the questionnaire were used. This could explain the lower prevalence of pain and depression in the present studies compared to previous studies^3,4^, but the different prevalences should also be interpreted as a potential non-representativeness of the general stroke population which lowers the generalizability of the results. The use of RLS instead of the more commonly used NIHSS as a proxy for stroke severity was due to substantial missing NIHSS data in Riksstroke. However, level of consciousness has shown to be equivalent to the NIHSS in previous studies^18^. Furthermore, there is a lack of more detailed stroke-related variables such as cognitive function in the present study.

The present study did not investigate changes in self-experienced symptoms and RTW due to work-related factors. Work-related factors have been shown to affect RTW after stroke^29,30^, and sickness presenteeism seems to be associated with the type of workplace^23^. Including work-related factors in future research about RTW and changes in self-perceived health and symptoms after stroke could help deepen the understanding of the deterioration in general health, and pain between 1 and 5 years post-stroke in the RTW group that was shown in the present study.

## Conclusion

The majority of working-age people with stroke RTW, and the people able to RTW experience better general health, less pain, and less depression at both 1 and 5 years post-stroke compared with those not able to RTW. However, in contrast to the no-RTW group, the RTW group’s general health and pain deteriorated between 1 and 5 years. This indicates a need for continued follow-up and support to ensure a balance between work and health for people who RTW after stroke.

## Data Availability

The datasets analysed during the present study are not publicly available due to Swedish regulation (https://etikprovningsmyndigheten.se/for-forskare/vad-sager-lagen/) that state that data cannot be made available for more than what has been approved by the Ethical Review Board. The data are from different registries (Riksstroke, Statistics Sweden, the National Board of Health and Welfare, and the Swedish Social Insurance Agency) and can be made available upon reasonable request to each of the registry managers.
